# Real-World Evaluation of a Large-Scale Blended Care-Cognitive Behavioral Therapy Program for Symptoms of Anxiety and Depression

**DOI:** 10.1089/tmj.2021.0590

**Published:** 2022-10-07

**Authors:** Jocelynn T. Owusu, Pam Wang, Robert E. Wickham, Alethea A. Varra, Connie Chen, Anita Lungu

**Affiliations:** ^1^ Lyra Health, Burlingame, California, USA.; ^2^ Department of Psychological Sciences, Northern Arizona University, Flagstaff, Arizona, USA.

**Keywords:** Internet-based intervention, cognitive behavioral therapy, anxiety disorders, depression

## Abstract

**Introduction:**

Prior studies have supported the effectiveness of blended interventions for anxiety and depression; however, outcomes research of large-scale blended interventions for these conditions is limited.

**Objective:**

: To investigate the outcomes of scaled-up blended care (BC) cognitive behavioral therapy (CBT), a program that combined video-based psychotherapy with internet CBT, for symptoms of anxiety and depression.

**Materials and Methods:**

: Participants were 6,738 U.S.-based adults with elevated symptoms of anxiety (Generalized Anxiety Disorder-7 [GAD-7] ≥ 8) and/or depression (Patient Health Questionnaire-9 [PHQ-9] ≥ 10) at baseline who received BC-CBT as an employer-offered mental health benefit. The primary outcomes, anxiety (GAD-7) and depression (PHQ-9) symptoms, were routinely measured in the program. Recovery and reliable improvement in outcomes were calculated, and growth curve models evaluated change in outcomes during treatment and the effects of engaging in psychotherapy sessions on outcomes.

**Results:**

: On average, participants received treatment for 7.6 (standard deviation = 6.2) weeks. By the end of care, 5,491 (81.5%) participants had reliable improvement in either anxiety or depression symptoms; in addition, 5,535 (82.1%) fell below the clinical threshold for either anxiety or depression symptoms (i.e., recovered). Declines in anxiety and depression symptoms were statistically significant over the course of BC-CBT (both* p*'s < 0.01), with the rate of decline significantly decreasing for each outcome as treatment progressed (both* p*'s < 0.01). Each psychotherapy session completed was significantly associated with lower anxiety and depression symptoms during the week of the session and the subsequent week (all* p*'s < 0.01).

**Conclusions:**

: This real-world study provides evidence that scaled-up BC-CBT can be effective in the treatment of symptoms of anxiety and depression.

## Introduction

Anxiety and depression are common conditions with significant consequences, including reduced quality of life and disability.^1–3^ Anxiety and depression also result in high health care and societal costs in the United States.^3–5^ Significant increases in the prevalence of anxiety and depression symptoms,^6–8^ as well as in unmet mental health therapy needs,^8^ during the COVID-19 pandemic have further highlighted the critical importance of improving access to effective, scalable interventions for anxiety and depression symptoms.

A recent meta-review of mental health interventions offered through employers (e.g., employee assistance programs) reported strong evidence for cognitive behavioral therapy (CBT) to improve anxiety and depression symptoms.^9^ This is aligned with the large body of evidence supporting the efficacy or effectiveness of CBT,^10,11^ and internet-based CBT (iCBT),^12,13^ for the treatment of anxiety and depression. However, although iCBT has the potential to improve the accessibility of CBT,^14^ standalone iCBT has significant disadvantages such as high dropout rates and the potential of not identifying negative outcomes.^15–17^

Blended care (BC) interventions, including BC-CBT, which combine face-to-face CBT sessions with iCBT, have been proposed as a delivery approach that can capitalize on the benefits of both face-to-face and iCBT.^18,19^ The configuration of blended interventions varies, and BC-CBT programs can be designed to emphasize either the face-to-face component or the Internet-based intervention.^19^ The important benefits of BC-CBT include its ability to provide both support from a therapist during sessions and beneficial online content between sessions,^18^ in addition to personalized treatment that can address participants' individual treatment goals.^20^ Video-based BC-CBT, which delivers the face-to-face component of BC-CBT through video sessions, may further improve the accessibility of care.^20^

Despite their benefits and promise, implementing BC-CBT programs in real-world settings poses unique challenges.^21^ For example, digital mental health interventions are most commonly provided as a standalone program,^22^ thereby assigning digital content (i.e., treatment modules) automatically. Meanwhile, therapists can manually assign digital care components within BC-CBT; thus, barriers to their adoption among therapists must be overcome. Previous research found variability among therapists in their willingness to include computer-assisted therapy tools in treatment.^23^ In addition, successfully disseminating psychotherapy at a large scale requires addressing various challenges that can influence outcomes, including the delivery at scale of high-quality training,^24^ maintaining therapists' effectiveness over time,^25^ and preventing burnout among therapists.^26,27^

Therefore, despite the strong evidence base for the efficacy of CBT for anxiety and depression, outcomes evaluations of BC-CBT programs in real-world settings are important as the scale of such programs increases. The aim of this real-world study was to evaluate anxiety and depression outcomes of a video BC-CBT program that was provided as a mental health benefit by employers. Previous research of this BC-CBT program reported significant reductions in anxiety and depression symptoms across treatment.^28^ This replication study builds on that study by evaluating program outcomes in a larger, new sample of participants after the BC-CBT program was scaled up by increasing its count of therapists by more than 700% (from 47 to 382 therapists). This study also extends the prior study by examining the effects of the number of therapy sessions on symptoms of anxiety and depression during treatment.

## Materials and Methods

### STUDY DESIGN

This was a real-world, retrospective cohort study of a BC-CBT program, which was offered by Lyra Health partnering with Lyra Clinical Associates, to self-referring individuals, and their dependents, as a mental health benefit from their employers. The Palo Alto University Institutional Review Board has determined this analysis of de-identified data as not constituting human subjects' research. Informed consent was obtained from all participants.

### PARTICIPANTS

Participants began the BC-CBT program between September 3, 2019 and July 21, 2021. All participants, as well as the therapists, were based in the United States, and each of the four U.S. Census Bureau regions were represented in the sample (i.e., Northeast, Midwest, South, West).^29^ Exclusion criteria for the BC-CBT program were previously described.^28^ BC-CBT program participants above the clinical cut-off for elevated symptoms of depression (Patient Health Questionnaire-9 ([PHQ-9] ≥ 10) or anxiety (Generalized Anxiety Disorder-7 [GAD-7] ≥ 8) at baseline were eligible for this study (*N* = 7,331).^30,31^

**Fig. 1.**
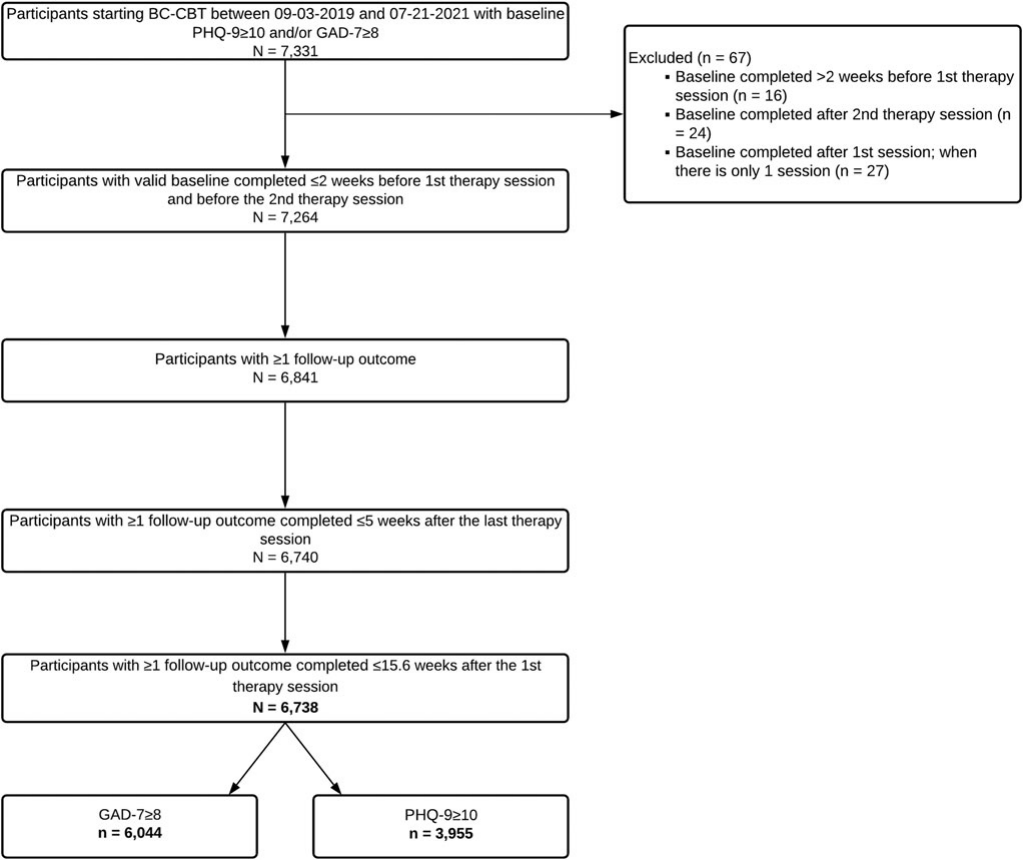
Participant flowchart. BC-CBT, blended care cognitive behavioral therapy; PHQ-9, Patient Health Questionnaire-9; GAD-7, Generalized Anxiety Disorder-7.

We excluded participants with baseline assessments collected >2 weeks before the first therapy session, after the second therapy session, or after the first therapy session when there was not a second therapy session (*n* = 67; 0.9%). We also excluded participants missing a valid second assessment (*n* = 423; 5.8%), and participants who only had post-baseline assessments that occurred >5 weeks after the last therapy session (*n* = 101; 1.4%). Finally, we excluded assessments if they were collected >15.6 weeks after the first therapy session (i.e., >1 standard deviation [SD] above the mean treatment duration). Two participants were excluded, because they had no valid follow-up data after this exclusion step. The final dataset included 6,738 participants (*Fig. 1*).

### TREATMENT

The BC-CBT program has been previously described in detail,^28^ but it can be summarized as combined live video-based sessions with a therapist plus between-session digital care tools (i.e., lessons and exercises, therapist feedback, assessments). Therapy sessions were conducted on a secure, proprietary HIPAA-compliant video platform developed by Lyra Health, and they were completely virtual. Therapy staff consisted of 382 licensed therapists (licensed clinical psychologists, licensed marriage and family therapists, licensed clinical social workers, or licensed professional counselors). This represents a 700% increase in the number of therapists from the previous BC-CBT evaluation, which included 47 therapists.

#### Training and quality assurance at scale

Therapists received >60 hours of intensive training that included a workshop, individual instruction, and self-study during the first month. Weekly video-based individual and group consultation was provided during the first 3 months, and the cadence reduced to bi-weekly thereafter. Due to COVID-19-related restrictions, all training was provided virtually. Therapy sessions were recorded with the participant's consent, and session content was reviewed on a quarterly basis by using an internal fidelity rating scale. Additional quality assurance included reviews of ongoing assessments, therapeutic alliance, documentation, and utilization.

#### Digital lessons and exercises

The digital treatment components were developed by Lyra Health based on transdiagnostic treatment approaches. Example principles and skills covered through the digital components include: clarifying values, understanding emotions, mindful awareness, cognitive restructuring, challenging avoidance (via behavioral activation and exposure), and communication skills. Therapists received alerts when clients completed assigned digital lessons and exercises, and they could send personalized feedback to clients. Therapists were also available via asynchronous messaging to answer questions, provide feedback, and clarify content.

### MEASURES

#### Anxiety and depression symptoms

As a part of BC-CBT, the PHQ-9 and GAD-7 were administered weekly, to monitor participants' depression and anxiety symptoms, respectively. Reliable improvement in anxiety and depression symptoms were measured by using metrics defined in previous research that identified decreases in the GAD-7 ≥ 4 and the PHQ-9 ≥ 6, respectively, as reflecting psychometrically reliable change.^24,32^ In addition, for each outcome, participants were identified as achieving recovery if their final scores were in the subclinical range (i.e., PHQ-9 < 10, GAD-7 < 8).

#### Other measures

Participants self-reported age, gender, and race and ethnicity via the online platform. Race and ethnicity was re-categorized as non-minoritized group (i.e., White), minoritized groups (i.e., Asian or Pacific Islander, Black or African American, Hispanic or Latino, Multiple, Other), or preferred not to disclose/missing (i.e., missing race and ethnicity data or responded “prefer not disclose”).

### STATISTICAL ANALYSES

Descriptive statistics were computed for the entire sample, and they were stratified by those with elevated symptoms of depression (i.e., PHQ-9 ≥ 10) or anxiety (i.e., GAD-7 ≥ 8) at baseline. Changes in anxiety and depression symptoms were examined by using paired-sample *t*-tests comparing the baseline and last available PHQ-9 and GAD-7 scores; these scores were also used to estimate the within-subjects effect sizes (Hedges' *g*).

The proportion of participants achieving reliable improvement and/or recovery was computed for the entire sample and across several subgroups: the “depression-only” subgroup (i.e., baseline PHQ-9 ≥ 10), the “anxiety-only” subgroup (i.e., baseline GAD-7 ≥ 8), and the “depression and anxiety” subgroup (i.e., participants with both PHQ-9 ≥ 10 and GAD-7 ≥ 8 at baseline).

A series of individual growth curve models (GCMs) were specified to evaluate changes in anxiety and depression symptoms trajectories over the course of BC-CBT. Model 1 included fixed-effects terms for linear (week) and quadratic (week^*2*^) time, which describe the average trajectory of symptoms, a random effect for the intercept across both participants (Level 2) and therapists (Level 3), and random effects for the linear and quadratic effects of time at Level 2. Model 2 retained Model 1 specifications and added a fixed effect for the time-varying covariate (TVC) describing the association between engagement in therapy sessions during the past 7 days and current symptom scores. For Model 3, the lagged effect of engagement in therapy sessions during the previous 8–14 days was added to Model 2 specifications. Analyses were conducted using R 3.6.3, including GCMs, which were fit by using the *lmer* library.

## Results

*Table 1* describes participants' characteristics. Participants' mean age was 33.23 years (standard deviation [SD] = 8.75). Overall, 4,378 (65%) participants were female, and 3,394 (50.4%) were from minoritized racial and ethnic groups. On average, participants received 6.00 therapy sessions (SD = 3.55), and they were in treatment for 7.58 weeks (SD = 6.20).

**Table 1.** Participant Characteristics

	ENTIRE SAMPLE		BASELINE DEPRESSION SYMPTOMS (PHQ-9 ≥ 10)	BASELINE ANXIETY SYMPTOMS (GAD-7 ≥ 8)
	*N* = 6,738		*N* = 3,955	*N* = 6,044
Age, mean (SD)	33.23 (8.75)	33.28 (8.89)	33.22 (8.69)
Gender, *n* (%)			
Female	4,378 (64.97)	2,548 (64.42)	3,937 (65.14)
Male	2,353 (34.92)	1,401 (35.42)	2,100 (34.75)
Missing	7 (0.10)	6 (0.15)	7 (0.12)
Race and Ethnicity, *n* (%)			
Non-minoritized Group			
White	3,048 (45.24)	1,707 (43.16)	2,717 (44.95)
Minoritized Groups			
Asian or Pacific Islander	1,761 (26.14)	1,050 (26.55)	1,579 (26.13)
Black or African American	349 (5.18)	220 (5.56)	309 (5.11)
Hispanic or Latino	599 (8.89)	375 (9.48)	549 (9.08)
Multiple	527 (7.82)	324 (8.19)	470 (7.78)
Other	158 (2.34)	90 (2.28)	142 (2.35)
Prefer not to disclose/missing	296 (4.39)	189 (4.78)	278 (4.60)
Baseline PHQ-9, mean (SD)	10.73 (5.04)	14.08 (3.53)	10.50 (5.19)
Baseline GAD-7, mean (SD)	11.90 (4.05)	12.22 (4.63)	12.66 (3.51)
No. of therapy sessions completed, mean (SD)	6.00 (3.55)	6.13 (3.66)	6.03 (3.58)
Duration of care (week), mean (SD)	7.58 (6.20)	7.71 (6.37)	7.61 (6.24)

GAD-7, Generalized Anxiety Disorder-7; PHQ-9, Patient Health Questionnaire-9; SD, standard deviation.

### RELIABLE IMPROVEMENT AND RECOVERY

Among participants with elevated depression symptoms at baseline (i.e., PHQ-9 ≥ 10), 3,279 (82.9%) had reliable improvement or recovery in depression symptoms by the end of care (*Table 2*). Similarly, among participants with elevated anxiety symptoms at baseline (i.e., GAD-7 ≥ 8), 5,151 (85.2%) had reliable improvement or recovery in anxiety symptoms by their final assessment. Of the entire sample with elevated symptoms of either depression (PHQ-9 ≥ 10) or anxiety (GAD-7 ≥ 8) at baseline, 5,964 (88.5%) participants had reliable improvement or recovery in either anxiety or depression symptoms by the end of care.

**Table 2.** Reliable Improvement and Recovery, ***n*** (%)

BASELINE SYMPTOMS	SAMPLE SIZE	RELIABLE IMPROVEMENT	RECOVERY	RELIABLE IMPROVEMENT AND RECOVERY	RELIABLE IMPROVEMENT OR RECOVERY
Depression symptoms (PHQ-9 ≥ 10)	3,955	2,759 (69.76)	3,115 (78.76)	2,595 (65.61)	3,279 (82.91)
Anxiety symptoms (GAD-7 ≥ 8)	6,044	4,761 (78.77)	4,500 (74.45)	4,110 (68.00)	5,151 (85.23)
Depression and anxiety symptoms (PHQ-9 ≥ 10 and GAD-7 ≥ 8)	3,261	2,055 (63.02)^a^	2,080 (63.78)^b^	2,453 (75.22)^c^	2,941 (90.19)^d^
Depression or anxiety symptoms (PHQ-9 ≥ 10 or GAD-7 ≥ 8)	6,738	5,491 (81.49)^e^	5,535 (82.15)^f^	4,960 (73.61)^c^	5,964 (88.51)^d^

^a^ Calculated as reliable improvement on GAD-7 and PHQ-9.

^b^ Calculated as recovery on GAD-7 and PHQ-9.

^c^ Calculated as reliable improvement and recovery on GAD-7 *or* reliable improvement and recovery on PHQ-9.

^d^ Calculated as reliable improvement or recovery on GAD-7 *or* reliable improvement or recovery on PHQ-9.

^e^ Calculated as reliable improvement on GAD-7 or PHQ-9.

^f^ Calculated as recovery on GAD-7 or PHQ-9.

### PAIRED-SAMPLE *T*-TESTS AND EFFECT SIZES

Those with baseline PHQ-9 ≥ 10 reported an average baseline PHQ-9 score of 14.08 (SD = 3.53) and a final mean PHQ-9 score of 6.07 (SD = 4.81), reflecting a statistically significant reduction (*M*_Difference_ = 8.01, SD_D_ = 5.34; *t*(3,954) = 94.37, *p* < 0.001, Hedges' *g* = 1.89). Among the participants with a baseline GAD-7 ≥ 8, the average baseline and end-of-care GAD-7 scores were 12.66 (±3.51) and 5.64 (±4.17), respectively, with a significant reduction in anxiety scores (*M*_Difference_ = 7.02, SD_D_ = 4.83), *t* (6,043) = 113.10, *p* < 0.001, Hedges' *g* = 1.82).

### GROWTH CURVE MODELING

#### Anxiety symptoms

Estimates of fixed effects and model fit statistics with GAD-7 scores as the outcome are provided in *Table 3*. Across all three models, there was a significant initial (linear) decline in GAD-7 scores (Model 3 *b* = −1.07, 95% CI: −1.11 to −1.04, *p* < 0.01), qualified by a significant quadratic effect (Model 3 *b* = 0.05, 95% CI: 0.05–0.05, *p* < 0.01), suggesting that the rate of decline in the GAD-7 scores diminished over the course of treatment.

**Table 3.** Growth Curve Modeling Results of Anxiety Symptoms (GAD-7), b (95% Confidence Interval)

	MODEL 1	MODEL 2	MODEL 3
Intercept	11.05 (10.95, 11.14)	11.37 (11.27, 11.47)	11.56 (11.46, 11.67)
	*t* = 228.18^***^	*t* = 225.85^***^	*t* = 225.64^***^
Week	−1.24 (−1.27, −1.22)	−1.19 (−1.22, −1.17)	−1.07 (−1.11, −1.04)
	*t* = −84.39^***^	*t* = −80.59^***^	*t* = −67.45^***^
Week^2^	0.06 (0.06, 0.07)	0.06 (0.06, 0.06)	0.05 (0.05, 0.05)
	*t* = 55.81^***^	*t* = 50.93^***^	*t* = 40.11^***^
Sessions past 7 days		−0.75 (−0.81, −0.69)	−0.86 (−0.93, −0.80)
		*t* = −24.35^***^	*t* = −27.67^***^
Sessions past 8–14 days			−0.63 (−0.69, −0.57)
			*t* = −19.41^***^
Log likelihood	−110,725.10	−110,432.80	−110,246.70
Akaike inf. crit.	221,472.30	220,889.60	220,519.40
Bayesian inf. crit.	221,567.20	220,993.20	220,631.60

^***^ *p* < 0.01.

In Models 2 and 3, the predicted TVC effect emerged, suggesting that completing a therapy session in the past week was associated with significant decreases in expected GAD-7 scores (Model 3 *b* = −0.86, 95% CI: −0.93 to −0.80, *p* < 0.01). In Model 3, a significant lagged effect emerged, suggesting that engaging with a therapy session 8–14 days earlier was associated with a significant decrease in GAD-7 scores (*b* = −0.63, 95% CI: −0.69 to −0.57, *p* < 0.01).

When demographic characteristics (i.e., gender, age, race and ethnicity) were incorporated into Model 3, none were significantly associated with GAD-7 scores. In addition, a likelihood ratio test confirmed that including demographic characteristics did not significantly improve the fit of Model 3 (χ^2^(4) = 6.47, *p* = 0.17).

#### Depression symptoms

Estimates of fixed effects and model fit statistics with PHQ-9 scores as the outcome are described in *Table 4*. Similar to the models for anxiety symptoms, across all three models there was a significant initial (linear) decline in PHQ-9 scores (Model 3 *b* = −1.25, 95% CI: −1.30 to −1.21, *p* < 0.01), which was qualified by a significant quadratic effect of time (Model 3 *b* = 0.06, 95% CI: 0.05 to 0.06, *p* < 0.01). In Models 2 and 3, each completed therapy session was significantly associated with a lower PHQ-9 score during that same week (Model 3 *b* = −0.95, 95% CI: −1.03 to −0.87, *p* < 0.01).

**Table 4.** Growth Curve Modeling Results of Depression Symptoms (PHQ-9), b (95% Confidence Interval)

	MODEL 1	MODEL 2	MODEL 3
Intercept	12.20 (12.07, 12.33)	12.56 (12.43, 12.70)	12.77 (12.63, 12.91)
	*t* = 181.46^***^	*t* = 179.71^***^	*t* = 179.75^***^
Week	−1.44 (−1.48, −1.40)	−1.39 (−1.43, −1.35)	−1.25 (−1.30, −1.21)
	*t* = −70.91^***^	*t* = −67.52^***^	*t* = −57.14^***^
Week^2^	0.07 (0.07, 0.08)	0.07 (0.06, 0.07)	0.06 (0.05, 0.06)
	*t* = 46.65^***^	*t* = 42.40^***^	*t* = 33.90^***^
Sessions past 7 days		−0.82 (−0.90, −0.74)	−0.95 (−1.03, −0.87)
		*t* = −20.40^***^	*t* = −23.18^***^
Sessions past 8–14 days			−0.68 (−0.77, −0.60)
			*t* = −16.15^***^
Log likelihood	−75,127.62	−74,922.73	−74,793.84
Akaike inf. crit.	150,277.20	149,869.50	149,613.70
Bayesian inf. crit.	150,367.60	149,968.10	149,720.50

^***^ *p* < 0.01.

Incorporating the lagged engagement predictor in Model 3 revealed that each therapy session completed during the prior 8–14 days was also associated with a lower PHQ-9 score (*b* = −0.68, 95% CI: −0.77 to −0.60, *p* < 0.01).

Turning to the demographic covariates, a marginally significant effect emerged for the unknown race and ethnicity category (*b* = 0.33, 95% CI: 0.01 to 0.65, *p* = 0.05), but all other demographic coefficients were non-significant. Results from a likelihood ratio test revealed that collectively, demographic covariates did not improve the overall fit of Model 3 (χ^2^(4) = 4.73, *p* = 0.32).

## Discussion

This study was a replication of a previous study that evaluated outcomes of the same BC-CBT program while including a smaller sample of participants (i.e., *N* = 385).^28^ Findings of the current study, which included more than 6,700 participants and was primarily conducted during the COVID-19 pandemic, were comparable to those of the previous, smaller-scale study that was conducted before the COVID-19 pandemic.

More specifically, anxiety and depression symptoms reduced significantly over the course of the treatment, and large within-subject effect sizes were sustained between the current study and previous outcomes evaluation of this BC-CBT program (depression symptoms *g* = 1.89 and anxiety symptoms *g* = 1.82 in the current study). In addition, the effects sizes of the current study were greater than those found in existing meta-analyses of CBT for depression (*g* = 1.19 for studies using self-report measures),^33^ and individual CBT/exposure therapy for anxiety disorders (*d* = 1.30).^34^ Similar to the previous study, the rates of decline in anxiety and depression symptoms decreased over time in the current study.

Also consistent with the previous study, more than 85% of participants fell below the threshold for clinically significant anxiety or depression symptoms, or had a reliable improvement in symptoms, by their final assessment in this study (i.e., 88.5% in the current study). Moreover, this study builds on the prior study by finding that each session of psychotherapy was independently associated with significantly lower anxiety and depression symptoms in the concurrent and subsequent week of the session. Overall, this large study provides evidence that clinical outcomes can be maintained after the scale-up of a BC-CBT program for anxiety and depression symptoms.

Although numerous studies have found evidence of the effectiveness of BC interventions for the treatment of anxiety and depression,^19,35,36^ there has been limited real-world research on the outcomes of large-scale BC-CBT programs for anxiety and depression. One naturalistic study of BC-CBT for anxiety and depression symptoms implemented in a mental health center found that those who received BC (i.e., Internet modules combined with face-to-face sessions) experienced significant improvements in global (i.e., social, psychological, occupational) functioning post-treatment and had comparable outcomes to those who received face-to-face care only.^21^ However, in that study, in addition to online sessions, BC participants with depression or anxiety received an average of 16.7 and 12.2 face-to-face sessions, respectively; this was similar to the amount received by those who had face-to-face care only. Meanwhile, in the current study, participants only received six psychotherapy sessions, on average.

Thus, the current study shows that BC-CBT can be effective in a real-world setting with a shorter number of sessions with therapists. However, it is of note that the current study found significantly greater reductions in symptoms of anxiety and depression, with each increasing number of psychotherapy sessions completed. Although prior research of this BC-CBT program found that greater engagement with digital video lessons was also associated with lower symptoms of anxiety and depression,^37^ there are limited studies of how the different components of blended interventions contribute independently and synergistically to clinical outcomes.^38^ Additional research in this area can inform the development of future BC interventions.

The current study has potential implications for improving public mental health, especially in light of a significant increase in anxiety and depression symptoms,^6–8^ and unmet mental health needs,^8^ with the COVID-19 pandemic. With 88.5% of participants achieving reliable clinical improvement or recovery in an average of six psychotherapy sessions, which is fewer sessions than most standard CBT interventions,^10,11,18^ the blended mental health care model may be a potentially more effective approach to scale mental health treatment within the context of the COVID-19 pandemic.^39^

Further, BC-CBT that is delivered completely virtually is uniquely positioned to improve mental health care accessibility during the pandemic. However, although the transdiagnostic approach used in this blended intervention has shown effectiveness for anxiety and depression, BC digital components can also be designed to target specific symptoms of mental health disorders (e.g., disordered eating, trauma, insomnia). Therefore, to successfully advance wider adoption of blended intervention, there is a need for further research on ideal candidates for blended interventions and the factors that should inform treatment tailoring (e.g., severity, individual needs),^38^ as well as the most favorable balance of face-to-face sessions and Internet components.^19,38^

This study had numerous strengths. It was conducted in a large sample and relied on validated measures of anxiety and depression symptoms. In addition, this study replicated the methods of a previous study of this program, which allowed for an evaluation of how scaling up may influence program outcomes. This study also accounted for potential confounding attributable to individual therapist differences by adding this variable (i.e., therapist) as a random effect to the GCM.

Nonetheless, this study had several limitations. Because this study did not use a randomized controlled trial design, results cannot be used to derive conclusions about causality. In addition, although outcomes measures were validated, anxiety and depression diagnoses were not confirmed by using the gold standard clinical interview. Further, 8% of eligible participants were excluded because of missing/invalid data. However, although there is not a definite cutoff for substantial data missingness, this falls below an acceptable cutoff of 10%.^40^ Moreover, because this intervention was only available to those who were employed or a dependent of an employee, findings may not be generalizable to the general population. Finally, because this study focused on outcomes during treatment and at the end of care only, the durability of outcomes beyond the end of care is unknown.

## Conclusions

Regular outcomes evaluations of large-scale psychological interventions are important to ensure that their effectiveness is maintained as programs change and grow over time, especially in the midst of global events that impact mental health on a large scale such as the COVID-19 pandemic. To the authors' knowledge, this real-world U.S.-based study is the largest to date to provide supporting evidence that the BC-CBT can be scaled up as a mental health benefit and preserve beneficial outcomes.

Additional program evaluation research on the longer term outcomes of BC-CBT for anxiety and depression symptoms is warranted. Further, as BC-CBT programs increase in scale, additional research on the most potent components of such programs is needed, to maximize effectiveness and efficiency.
